# Integrated Monitoring of *Mola mola* Behaviour in Space and Time

**DOI:** 10.1371/journal.pone.0160404

**Published:** 2016-08-05

**Authors:** Lara L. Sousa, Francisco López-Castejón, Javier Gilabert, Paulo Relvas, Ana Couto, Nuno Queiroz, Renato Caldas, Paulo Sousa Dias, Hugo Dias, Margarida Faria, Filipe Ferreira, António Sérgio Ferreira, João Fortuna, Ricardo Joel Gomes, Bruno Loureiro, Ricardo Martins, Luis Madureira, Jorge Neiva, Marina Oliveira, João Pereira, José Pinto, Frederic Py, Hugo Queirós, Daniel Silva, P. B. Sujit, Artur Zolich, Tor Arne Johansen, João Borges de Sousa, Kanna Rajan

**Affiliations:** 1 CIBIO-InBIO, Centro de Investigação em Biodiversidade e Recursos Genéticos, Campus Agrário de Vairão, Universidade do Porto, 4485-661 Vairão, Portugal; 2 Department of Chemical & Environmental Engineering/Underwater Vehicles Laboratory, Universidad Politécnica de Cartagena (UPCT), Alfonso XIII, 52, E-30203, Cartagena, Spain; 3 Centre for Marine Sciences (CCMAR), University of Algarve, 8005-139 Faro, Portugal; 4 Faculdade de Engenharia da Universidade do Porto, Rua Dr. Roberto Frias, 4200-465 Porto, Portugal; 5 Oceanscan-MST, Avenida Liberdade, Polo Mar UPTEC, 4450-718 Matosinhos, Portugal; 6 Center for Autonomous Marine Operations and Systems, Department of Engineering Cybernetics, Norwegian University of Science and Technology, Gløshaugen, Trondheim, Norway; 7 Marine Biological Association of the United Kingdom, The Laboratory, Citadel Hill, Plymouth PL1 2PB, United Kingdom; 8 IIIT Delhi, New Delhi, 110020, India; Universitat Bielefeld, GERMANY

## Abstract

Over the last decade, ocean sunfish movements have been monitored worldwide using various satellite tracking methods. This study reports the near-real time monitoring of fine-scale (< 10 m) behaviour of sunfish. The study was conducted in southern Portugal in May 2014 and involved satellite tags and underwater and surface robotic vehicles to measure both the movements and the contextual environment of the fish. A total of four individuals were tracked using custom-made GPS satellite tags providing geolocation estimates of fine-scale resolution. These accurate positions further informed sunfish areas of restricted search (ARS), which were directly correlated to steep thermal frontal zones. Simultaneously, and for two different occasions, an Autonomous Underwater Vehicle (AUV) video-recorded the path of the tracked fish and detected buoyant particles in the water column. Importantly, the densities of these particles were also directly correlated to steep thermal gradients. Thus, both sunfish foraging behaviour (ARS) and possibly prey densities, were found to be influenced by analogous environmental conditions. In addition, the dynamic structure of the water transited by the tracked individuals was described by a Lagrangian modelling approach. The model informed the distribution of zooplankton in the region, both horizontally and in the water column, and the resultant simulated densities positively correlated with sunfish ARS behaviour estimator (r_s_ = 0.184, *p*<0.001). The model also revealed that tracked fish opportunistically displace with respect to subsurface current flow. Thus, we show how physical forcing and current structure provide a rationale for a predator’s fine-scale behaviour observed over a two weeks in May 2014.

## Introduction

Satellite remote sensing of the marine ecosystem has become an important ecological instrument for both environmental monitoring and conservation assessments [1]. Critically, systematic satellite imagery has been used to better understand and characterise the movement and habitat of marine species. Different studies have made use of remote sensed imagery to integrate marine species’ tracks and reveal important spatial dynamics’ and their environmental drivers (e.g. [2,3,4]). Typically these tracks are also obtained with the help of satellite-based tracking systems. However, challenges remain with remote sensing, which besides being restricted to the monitoring the sea surface, also has significant limitations in coastal. Coastal regions are fundamental for several marine species that use them as nurseries, moving corridors and/or foraging areas [5]. However, remote sensing of coastal habitats remains problematic for the following reasons: a) spatial resolution fails to capture smaller coastal features; b) approximations and corrections of pixel-based data in highly-mixed coastal areas leads to inaccuracies; c) the change in temperatures in shallow waters which remote sensing's resolution fails to capture; and, d) land/sea interfaces comingle pixels with mixed water/land temperatures (for instance in [6]). Thus, although proven useful and essential in oceanography, remote sensed features in coastal areas need substantial improvement to allow comprehensive studies in marine ecology.

Conversely, coarse-scale resolution associated with the remote sensed environment (>1 km) is lower than that currently attained by new satellite tracking systems (i.e. Fastloc GPS, with 50% of locations within 18 m and 95% within 70 m of the true position) [7]. Hence, both environmental integration of behavioural patterns and habitat use are still hindered by the low accuracy associated with satellite retrieved environmental features. For marine species, satellite tracking has allowed the description of not only animal movement patterns’, spatial distribution and behaviours, but has also highlighted important interactions with the ecosystem [8,9]. A major breakthrough was the advent of the Fastloc-GPS^™^ tracking system, which reduced the spatial errors associated with obtained positions [~10 km—Argos [10] or ~100 km—Light-level [11] to less than 60 metres [7,12,13]]. The high accuracy of GPS-based geolocation has proven invaluable in revealing both behavioural patterns of less well-known species and relationships between animals and the surrounding environment [14–16]. One such example was the Fastloc-GPS^™^ tracking of ocean sunfish (*Mola mola*, Linnaeus 1758), in the Gulf of Cadiz [13]. High accuracy GPS geolocated tracks allow analysis of areas of restricted search (ARS) [13] along a tracked path, which helps in identifying the regions where animals allocated more time, presumably to forage. Critically, patterns of a species movement usually reveal the preferred habitats, critical areas for breeding, feeding and for protection or shelter [8,17]. While currently available tracking systems have improved marine species spatial dynamics characterisation, further integration of fine-scale movements in the habitat are still needed. Importantly, the understanding of the environmental integration of remotely retrieved trajectories will improve our knowledge of animal movement patterns, as predators alter their behaviour in accordance to the spatial-temporal distribution of resources [18,19].

One way to improve knowledge of coastal environments or to increase the resolution at which the environment explored by an organism is sampled, is with the use of robotic platforms such as autonomous underwater vehicles (AUVs) [20,21]. AUVs enable longer-term, systematic broad area coverage at finer scales of the upper water column. This includes high-resolution observations of the coastal bio geophysical ocean processes [22–24], coastal circulation [25], eddy monitoring [26], phytoplankton variability [27] and/or zooplankton [28] assessments. The Light AUV (LAUV) [29] in particular, has become a workhorse for a range of oceanographic applications and is supported by advanced command and control software that allows a user from ship or shore (or even remotely via satellite communications) to monitor its environment and its own state and operational effectiveness. Hence, AUVs provide important water-column properties to augment and ground-truth remote sensed data.

Theoretical links between the environment (e.g. water temperature, currents, primary productivity) and species behaviour are usually established through combined approaches, including remote sensing and tracking systems. One such methodology are Lagrangian models, which have been used to study the behaviour of low swimming capacity organisms at fine-scales [30–32]. These models work by releasing virtual particles in a simulated field of oceanic currents with the temporal and spatial progression of the particles tracked in space and time. Simulating the displacement of particles in a hypothetical current field, accurately informed by high-resolution satellite imagery, and validated by *in situ* data collections, allows a hydrodynamic model to be designed and analytically described for a specific region. Hence, the spatial aggregation of particles reflects behavioural patterns, expressed in terms of encounter probabilities of such organisms at sea. Ultimately, both environmental monitoring and such oceanographic models have progressed our understanding of the marine ecosystem.

The novelty of this work is in bringing together the analysis of the high-resolution movements of sunfish with the use of Lagrangian models coupled with remote sensing and *in situ* observations while supported by video analysis of encountered current driven particles, was used as proxy for zooplankton. Our objectives are multi-fold. First, we aim to characterise the factors associated with sunfish behaviour by estimating the environmental drivers for both predator behaviour and prey abundance, at fine scales of resolution. We do so by analysing high-resolution behavioural patterns, namely foraging *versus* travelling, and simultaneously integrating the *in situ* zooplankton densities in the environment. Second, we aim to provide a proof of concept of the utilisation of autonomous robots towards continuously monitoring a habitat occupied by a pelagic predator by bringing to bear advanced tools for command and control of robotic vehicles, hitherto never deployed for such environmental monitoring. Third, we present a Lagrangian modelling approach, which was calibrated and validated with *in situ* collected ADCP data and drifters’ tracks. Finally, in bringing together such techniques, we demonstrate a viable set of methods for enabling inter-disciplinary science and engineering in the field including using such experiments as pedagogical tools for young researchers.

## Material and Methods

**Fig 1:**
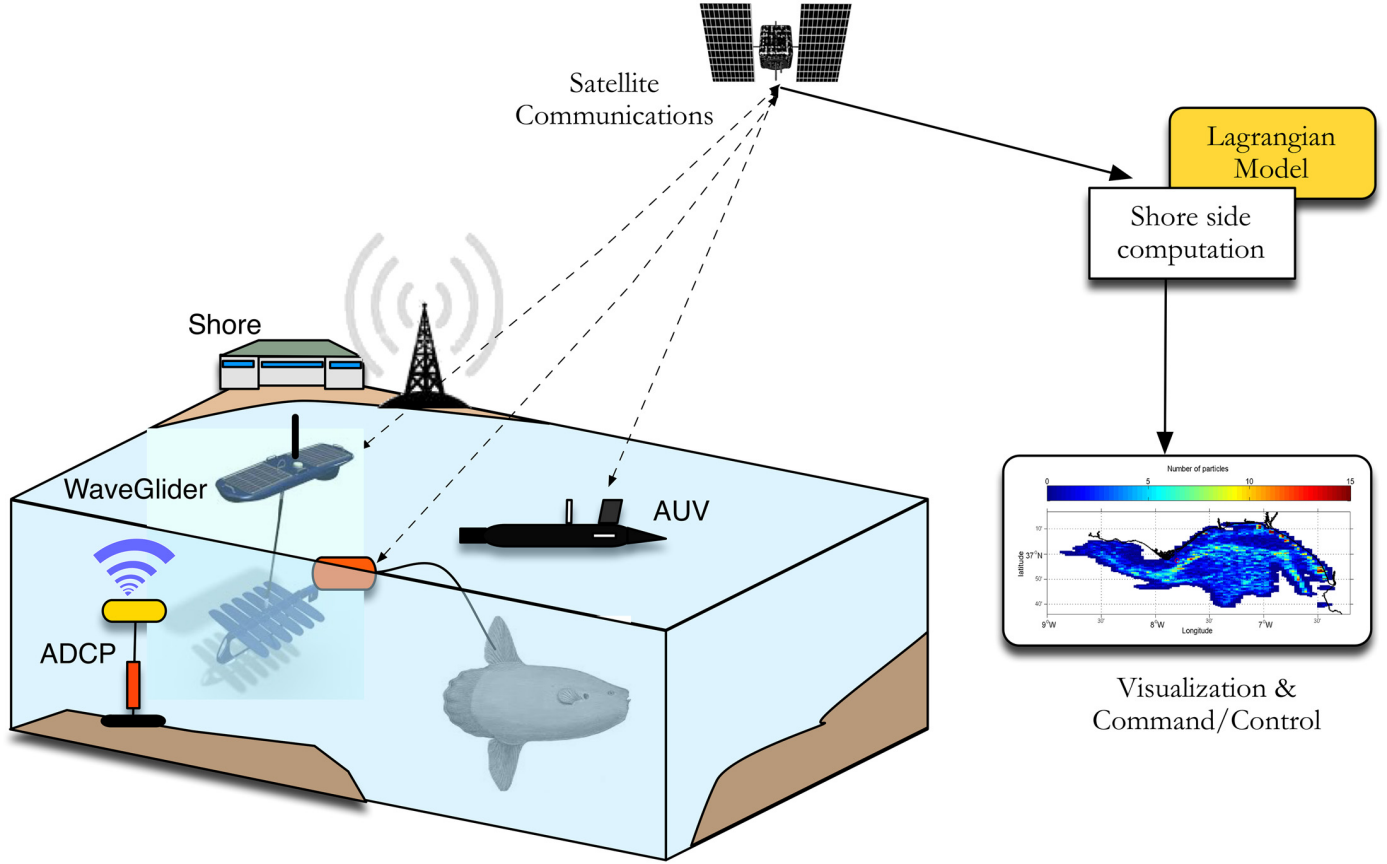
Schematic experiment diagram with tagged sunfish, ADCP (Acoustic Doppler Current Profiler), the WaveGlider ASV (Autonomous Surface Vehicle) and AUV (Autonomous Underwater Vehicle).

**Fig 2:**
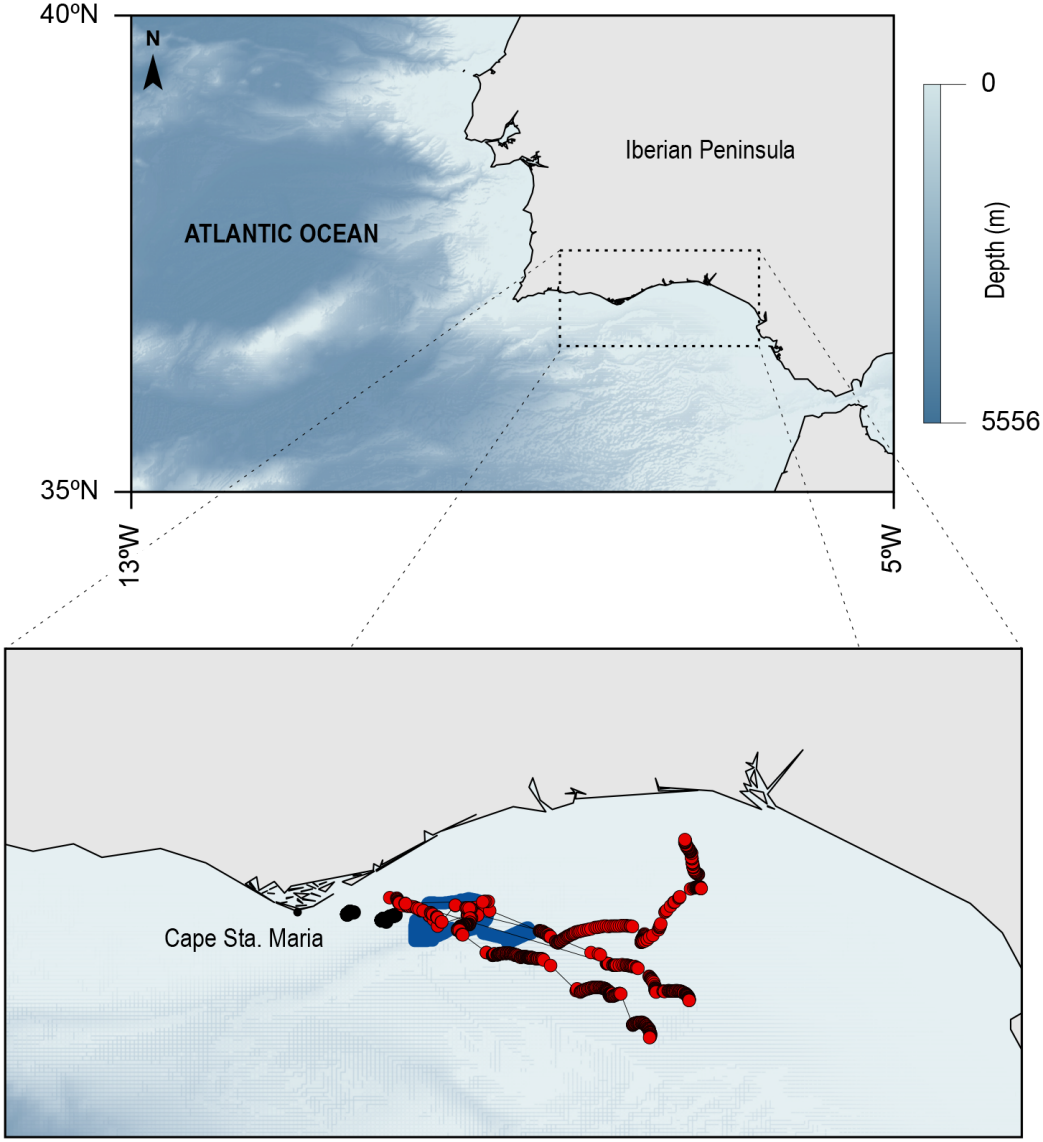
Map defining the study region, SW Iberian, including the northern margin of the Gulf of Cadiz and the Strait of Gibraltar. Bathymetric chart noted by the blue bar. Detailed tracks recorded in this study are represented in the inset and all tracks recorded (AUV—black; WaveGlider—blue and sunfish GPS—red dots).

The present study results from the integration of different technologies to study the behaviour of sunfish and its relation with the immediate environment at fine-scales (Fig 1). Data collected from autonomous underwater vehicles, an inshore ADCP and satellite tags attached to sunfish were collated for the near-real time description of both fish trajectories and water column characteristics. The target region of our experiment is located east of Cape Sta. Maria, in the northern margin of the Gulf of Cadiz, in southwest Portugal (Fig 2). The region is dominated by an intense frontal activity, associated with upwelling and relaxation events.

### Satellite tags

In this study, we used custom-made tags to track fish movements. The tags were based on the hardware of the Spot Personal Tracker (SPOT—https://www.findmespot.com/en/). This transmitter provides an accurate position (<10 m) due to its GPS receiver module. The tags will be referred to as SPOT-GPS throughout the paper. When at the surface, a water switch turns on the tag in *tracking mode* which automatically attempts to send its position every 10 minutes for 24 hours. The SPOT-GPS tag was especially conceived to provide the high performance and resolution of GPS tracking systems used in marine tracking studies (e.g. Fastloc-GPS [7]) at low costs. Both tag design and specifications are provided in the S1 Text and in [33].

### Sunfish tagging

A total of 10 individuals were successfully GPS tagged in a set-net off southern Portugal, in May 2014. Here, dozens of sunfish enter passively and are released from the set-net on a daily basis. To reduce tagging stress, all individuals were tagged underwater, and tags were attached externally via a 1.5 m monofilament tether tested to 200 lb stress and attached with alloy crimps at the base of the caudal fin [13,34,35]. This attachment assured that the tags were towed by the fish while travelling and the chosen length of filament maximised the probability of the tag breaking the surface to transmit every time the sunfish remained at the surface. This study was performed in national waters according to national Portuguese laws for the use of vertebrates in scientific research (Directive 113/2013, Ministério da Agricultura, do Mar, do Ambiente e do Ordenamento do Território), and all procedures followed the EU legislation Directive 2010/63/EU. Our study did not involve endangered or protected species, no animals were sacrificed, and procedures for reduction, replacement and refinement were thoroughly undertaken. Moreover, tagging was performed whilst adopting preventive mitigation techniques, such as the thoroughly sterilize of the material to minimize the potential infection risk and ensuring a predictable and relatively brief wound healing, with the procedure having a trivial impact to the animal.

### Tracking analysis—ARS

Retrieved GPS locations were processed to clean erroneous positions (e.g. those found to be on land). Given the small associated error, all sunfish GPS tracks were then analysed using track-shape metrics, which were used to inform the along-the-track behavioural states and thus define areas of restricted search (ARS). Hence, time spent within a specific area along the path informs the search effort, with increased time and less straight line movements, characteristic of a search behaviour. Briefly, speed over ground was computed between consecutive positions; sinuosity, defined as the ratio between an individual’s total displacement over three days and the straight-line distance between the positions on those three days, was also calculated informing path tortuosity; and, lastly, First Passage Time (FPT) [36] was applied to all tracks, selecting the peak in log variance and assigning the respective value to all fish positions. Lastly, ARS was defined as the combination of at least two of these three parameters (speed, sinuosity and FPT), in such ways that consecutive sunfish positions within the first quartile of speed and sinuosity, or the last quartile of log FPT were assigned to be intense searching (ARS) [37].

### Environmental integration

#### Remote sensed data

To better describe the oceanographic setting of the region of the experiment along with the moored ADCP data, composite images at 4 km spatial resolution of the Sea Surface Temperature (SST) were acquired from the NASA’s MODIS (Moderate Resolution Imaging Spectroradiometer) Aqua satellite data platform PO.DAAC (http://podaac.jpl.nasa.gov/dataaccess), covering four periods (16^th^– 18^th^ May, 21^st^– 23^rd^ May, 25^th^ May– 1^st^ June, 2^nd^-9^th^ June). Furthermore, Chlorophyll *a* (Chl *a*) remote sensing daily composites, at 4 km spatial resolution, were also extracted from the MODIS Level 3 NASA remote dataset from the NASA Ocean Biology Processing Group (OBPG—http://oceancolor.gsfc.nasa.gov) for the periods covering the deployment. The maps were inspected to infer surface patterns and frontal regimes in the Gulf of Cadiz at the time of the experiment.

#### Thermal gradients and coastal fronts

Remote sensed daily SST maps were used, in R (programming) language script, to compute both 3 and 5 day averaged maps. For each temporal resolution, gradients of temperature at a 3 x 3 pixel resolution (12 km) were calculated to inform persistent thermal features. Furthermore, using the Marine Geospatial Ecology Tool in ArcGIS MGET—[38] daily SST images were rasterized and Cayula-Cornillon fronts [39] identified at different thresholds (0.4, 0.6 and 0.8). Following the procedure in [40] these frontal gradients (Fmean) were then multiplied by the average fronts depicted in cloud free observations (Pfront) to compute front persistence metrics (Fpersist). Lastly, final average frontal persistence was calculated by assigning different weights to the front persist values obtained with the different thresholds (0.25*Fpersist 0.4 + 0.5*Fpersist 0.6 + 0.75*Fpersist 0.8).

#### Integration of sunfish behaviour on the environment

To investigate the spatial relationship between tracked sunfish positions and the immediate environment, all four remotely sensed daily variables (SST, SST gradients, Chl *a* and front metrics) were extracted for each retrieved position. This way, both travelling and ARS modes along the individual tracks were environmentally characterised and tested (using the *t* test) which provided evidence for specific drivers for switching behavioural states.

#### Autonomous vehicles

The LAUV's used for this experiment were equipped with a calibrated XR-620 CTD from RBR (https://rbr-global.com/), a fluorometer and an externally mounted GoPro^®^ camera to record ambient conditions in clear waters. These vehicles can dive to a depth of 100 meters and carry enough battery power to stay in the water in excess of 24 hours, while traveling at 1 ms^-1^ speed over ground. For localization, the LAUVs use GPS at the surface and an Inertial Navigation System (comprised of a compass, accelerometers and gyroscopes) for underwater navigation. In order to communicate to shore, the vehicles use Wi-Fi or GSM/HSDPA communications when at the surface or an acoustic modem while underwater for sparse messaging. The latter is typically used to track the vehicle and to receive near real-time updates when it is surveying the water column. For offshore operations these vehicles were also fitted with an Iridium satellite modem. Advanced software for planning, visualization, control, data archival and analysis with networked connectivity bridged the tasks spread across various phases of the experiment. The Neptus desktop software [41] provides the situational awareness, planning and data archival services with an elaborate graphical front end. Typically, the user loads maps of the operational area with detailed bathymetric and surface obstructions (if any) and generates a mission. For this experiment, Neptus provided a visual front end to an Artificial Intelligence based control system embedded on the LAUV which can plan and execute high level directives provided by a user on ship or shore. Details of this controller are outside the scope of this manuscript and are available in [42,43]. In addition to AUVs, an autonomous surface vehicle (ASV), the WaveGlider (WG) was used during the experiment. The WG is a two-body wave-energy powered autonomous surface platform from Liquid Robotics. The WG carries a range of sensors including an ADCP, a pumped CTD, a complete weather station to measure atmospheric conditions including ambient temperature and wind speed and direction and an AIS receiver.

#### Video recording of AUV tracks

GoPro^®^ cameras were attached to the AUVs, enabling video recording of the transiting environment within the water column. Image processing techniques were used on the recorded images to detect and quantify drifting particles that were of interest. The image processing procedure consisted of applying (a) background subtraction to a selected region of interest (ROI) (b) Gaussian Mixture of Normal Distributions and (c) sequential contour detection to determine the number of particles detected per image. Briefly, the procedure (a) is applied to reduce the image size to the desired area of interest. The change in illumination of the ocean waters and temporal background as the AUV transits requires the image processing unit to learn background change continuously [44]. For this purpose, a Gaussian Mixture of Normal Distributions as described in [45] was used to model a multimodal sequence of background images by considering each pixel's normal distribution on its background mixture corresponding to the probability of observing a particular intensity on the pixel. This procedure is carried out in (b). After (b), erode and dilate morphological operations are carried out on the image to illuminate the particles and determine their contours in procedure (c).

We found that recorded particles were a) larger than 1 mm in diameter, given the resolution of the GoPro^®^ camera and image processing techniques; b) not just drifting with the water motion, as they changed their forms slightly from one frame to the other; and, c) mostly low density particles with translucent tissues. Hence, and based on the number of contours detected, the suspended particle density is determined per video frame and used as a proxy for zooplankton abundance in the region.

#### Integrating zooplankton with the environmental data

To contextualise the physical environment for variations in particles density (a proxy for zooplankton), the recorded densities were further analysed in relation to *in situ* temperature and depth measurements recorded by AUVs. In summary, for each AUV survey, both particle counts and recorded temperatures were averaged per second. These were then analysed along the vehicle track and geolocated. Finally, the recorded zooplankton variation (counts per second) was investigated in relation to the same environmental drivers found to explain sunfish behaviour (SST, thermal gradients, Chl *a* and SST front metrics—strength and persistence). Extracted features related to each AUV position were correlated using Pearson correlation coefficient, with their respective particle abundance.

### Modelling methods

#### Hydrodynamics

A daily 3D map of velocity current components (eastward and northward) in the area during the time span of the experiments (25^th^ May to 1^st^ June 2014) was obtained from Copernicus Marine Environment Monitoring Service (CMEMS—http://marine.copernicus.eu/). The Atlantic-Iberian Biscay Irish- Ocean Physics Analysis and Forecast (IBI MFC) system within CMEMS, based on the Hydrodynamic model NEMO v3.4, provided a 5-day hydrodynamic forecast derived by meteorological and oceanographic forcing. The NEMO model solves the three-dimensional finite-difference primitive equations in spherical coordinates discretized on an Arakawa-C grid and 50 geopotential vertical levels (z coordinate). The grid, initial and lateral open boundary conditions (temperature, salinity, current velocities and sea level) are provided by the Operational Mercator Global Ocean Analysis and Forecast System [46]. The model provides output as daily mean data with a spatial resolution of 2 x 2 Km.

#### The Lagrangian model

The Lagrangian model was written in Octave (https://www.gnu.org/software/octave/) and was fed with the 3D eastward and northward current velocity components obtained from the Copernicus hydrodynamic forecast system. As the field of currents obtained from the CMEMS was a daily mean average, the particles in the Lagrangian model were released at a specific position and left to run (displace) for an hour of simulation. A second location after the first time step, was then used as the starting location with currents at that time and position, forcing the particle displacement iteratively for successive time steps. For each time step, the current components and the particle positions were obtained and multiplied by a random number, uniformly distributed between 0 to 2, to account for the influence of turbulence. The simulated domain had a grid cell of 2 km x 0.6 km in longitude × latitude with a time step of 1 hour. Two kinds of Lagrangian simulations were performed after running several tests to optimize the ratio between computational performance and model capabilities. The first to determine whether the sunfish followed the currents, or would stay in areas against it, whilst the second to find where particles aggregate to investigate if the tagged fish movements matched those patterns. In the first, releasing 200 particles at the beginning of each fish track (first position recorded for each day), and leaving them washed away during the time the fish was in surface, was enough to check whether the sunfish tracks overlapped current motion. By superimposing these particles distribution maps produced by the Lagrangian model with the sunfish track we were able to detect whether or not the fish were following the directionality of the currents. In the second, releasing 8100 particles regularly distributed in the entire domain provides a consistent distribution pattern after 48 hours. These distribution maps can be interpreted as encounter probability maps for fishes to find a particle on their way.

Various sources were used to validate both the hydrodynamic outputs of the CMEMS, and the Lagrangian model. The CMEMS outputs were validated against ADCP data recorded—Northward (*r* = 0.99, pbias = 22) and Eastward components (*r* = 0.75, pbias = 20) mounted on the WaveGlider, operating in the area (description in Section 5). The Lagrangian model was validated against satellite tracked drifters (n = 3) deployed in the area. Details of model validation are in S1 Text.

#### Currents

To better understand the oceanographic settings in the study area, east of Cape Sta. Maria, in the Gulf of Cadiz, *in situ* data were collected by two bottom mounted ADCP along the 23 m depth isobaths of Armona (37° 00.648’ N; 007° 44.480’ W—ADCP Workhorse 600 kHz, TRDI) and Tavira (37° 04.693’ N; 007° 36.505’ W—ADCP Sentinel V50 500kHz, TRDI); temperature at the ADCP, 1.5 m above the sea bottom, was also recorded. Data processing included a quality check, removal of the cells near the boundaries affected by reflections, re-interpolation of the velocities into 0.5 m bins along the water column, rotation of the velocity of valid cells in, along and cross-shore components according to the angle of maximum variance, and finally low pass filtering of the data with a Butterworth filter at 40 h cut-off period to remove tidal and small scale perturbations. Analysis of the data revealed useful information to understand the oceanographic frame of the experiment while also validating the *in situ* autonomous vehicles sampling and Lagrangian model calibrations.

## Results

### Tracked sunfish

Of the 10 individuals tagged, four (ranging in sizes from 0.52 to 0.79 m) successfully reported positions between 2–6 days (Table 1, Fig 2 and all tracks can be found in Movebank.org). Overall, six SPOT-GPS tags failed to transmit and fish were tracked on average for 4.25 days (± 1.7 standard deviation) and a mean of 35.6 positions were retrieved daily (±17 standard deviation), ranging from 16 up to a maximum of 52 locations per day.

**Table 1:** Summary of successfully SPOT-GPS tagged and tracked sunfish in this study.

Tag ID	Tagging date	Last position	Days at sea	Positions	Positions per day
17	05-05-2014	10-05-2014	5	240	48.0
13	12-05-2014	14-05-2014	2	33	16.5
07	12-05-2014	18-05-2014	6	314	52.3
08	12-05-2014	16-05-2014	4	102	25.5

### Environmental integration of sunfish tracks

**Fig 3:**
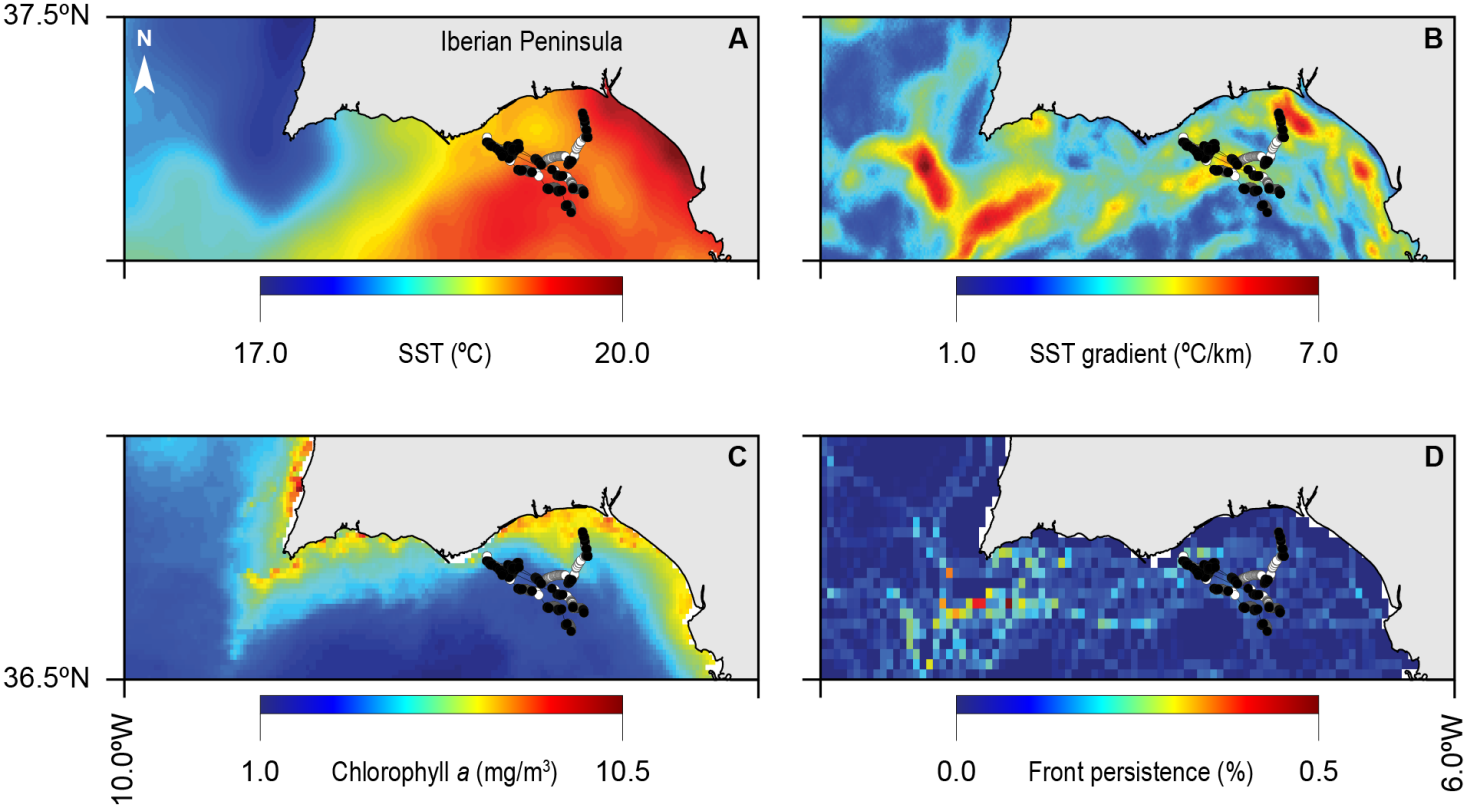
Integration of sunfish trajectories (black: ARS, white: travel) with the environmental variables A) SST, B) SST gradients, C) Chl *a* and D) SST fronts. Images are composites for each environmental feature, covering the entire tracking period of 14 days [5^th^ to 10^th^ and 11^th^ to the 20^th^ of May, 2014].

Different remote sensed environmental features were extracted for each sunfish position including SST, SST gradients, Chl *a* and front metrics. These were then used to explain sunfish behavioural patterns by testing for significant differences between each of the individual behavioural phases (ARS versus Travel, see Table 2). Fig 3 shows a map of all SPOT-GPS sunfish tracks overlaid on each of the four environmental variables’ composite averaged maps for the entire tracking period.

**Table 2:** Descriptive statistics of the encountered environment. Mean and standard deviation (s.d.) for each environmental feature are presented.

	SST	SST gradients	Chl *a*	Fronts
**ARS**	19.348 (0.324)	0.428 (0.262)	0.384 (0.447)	0.413 (0.237)
**Travel**	19.361 (0.367)	0.366 (0.253)	0.494 (0.705)	0.353 (0.247)

Overall, sunfish behaviour was found not to be directly influenced by SST as no significant differences were found between the thermal conditions encountered by the fish when travelling or when in ARS (*t* test = 0.48, *p* = 0.628, df = 651). However, a significant influence of SST gradients was found in sunfish behaviour, with ARS mode being detected in sharp variations of temperature when compared to travelling (*t* test = -3.08, *p*<0.05, df = 592). Unexpectedly, ARS was found to occur in areas with significantly lower primary productivity (using Chl *a* as a proxy) when compared to the travelling mode (*t* test = 2.53, *p<0*.*05*, df = 687). Lastly, thermal front intensity was found to be a significant driver for sunfish behaviour with higher occurrence of ARS in waters characterised by a sharp frontal region when compared to travelling modes (*t* test = -3.22, *p* = <0.05, df = 621). Hence, taken together, SST appears not to drive sunfish foraging behaviour, whereas both thermal gradients (front and slopes) were significantly higher in ARS when compared to travel modes. Lastly, sunfish ARS was linked to lower values of Chl *a*, the only proxy for primary productivity.

### Estimating zooplankton densities

**Fig 4:**
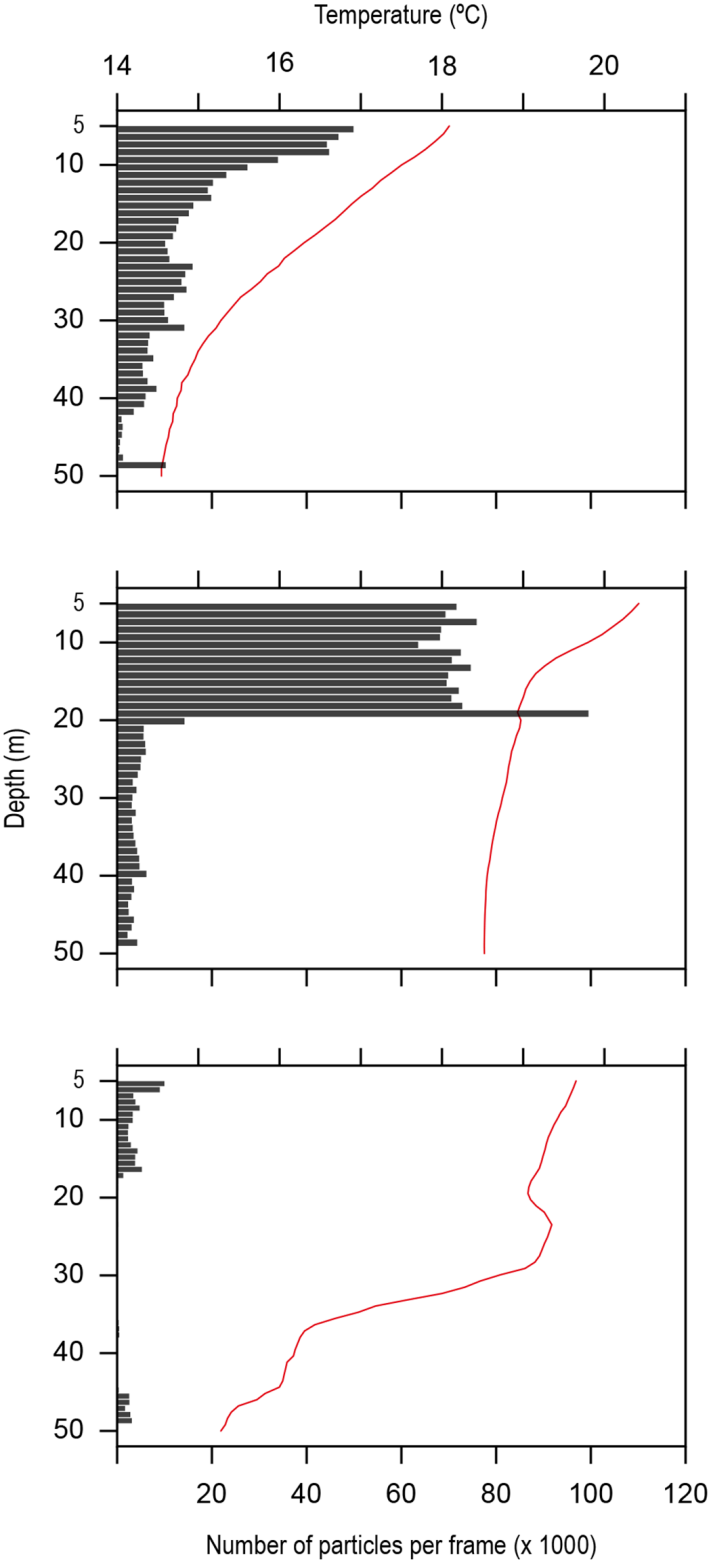
GoPro^®^ camera recorded zooplankton densities—number of particles detected per frame (grey bars on x axis) with depth (y axis) and simultaneous water column temperature records (line and top axis), for three different surveys on two separate days (18^th^ and 19^th^ May).

The AUVs were continuously sampling the water column for a total of 18 hours during six different surveys close in time to when fish were tracked (on 7^th^, 8^th^, 13^th^, 17^th^, 18^th^ and 19^th^ May) and near the frontal region. Of these, analysis of the video recordings for three surveys (n = 7 hours in total) revealed the presence of drifting particles (18^th^ and 19^th^ May, Fig 2 and S2 File). These particles were then analysed in relation to both *in situ* recorded temperatures and depth (see Fig 4 below). In waters with marked stratification the presumed zooplankton abundance decreased one order of magnitude (Fig 4C) when compared to well-mixed waters (Fig 4A and 4B). Independently of stratification, estimated zooplankton densities decreased significantly with increased depth (r_s_ = -0.534, *p*<0.001) and in contrast, we found a significant positive correlation with water temperatures (r_s_ = 0.439, *p*<0.001) represented as continuous lines in Fig 4.

Remarkably, negative correlations were found with remote sensed data for SST, Chl *a*, and front intensity for the video recorded particle abundance. On the other hand, both SST gradients and front distance were positively correlated with increased estimated zooplankton densities (see Table 3). Hence, as in the sunfish ARS mode, there is a strong influence of SST slopes in the abundance of zooplankton, whereas Chl *a* is a negative driver for both zooplankton richness and sunfish foraging behaviour.

**Table 3:** Correlation (Pearson) coefficients between estimated zooplankton densities and environmental features.

***SST***	*p <0*.*001; r*_*p*_ = *-0*.*061*
***SST Gradients***	*p <0*.*001; r*_*p*_ = *0*.*402*
***Chl a***	*p <0*.*001; r*_*p*_ = *-0*.*135*
***Front Value***	*p <0*.*001; r*_*p*_ = *-0*.*259*
***Front Distance***	*p <0*.*001; r*_*p*_ = *0*.*275*

### Lagrangian integration of zooplankton densities at the front

**Fig 5:**
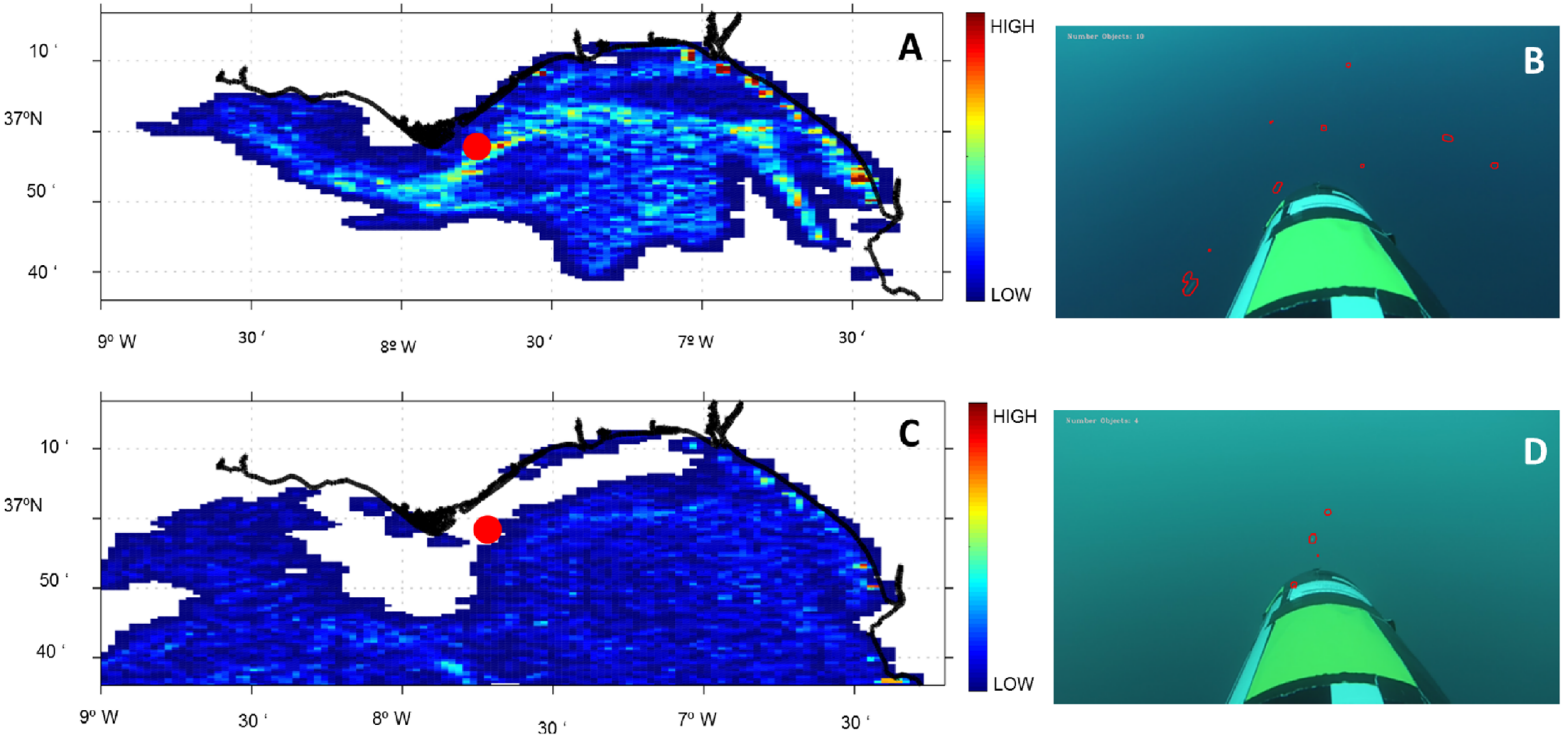
Lagrangian model particle concentration at subsurface (2 m depth) (A) for May 18^th^, (C) for May 19^th^. The red dot denotes the starting position for the particles simulation. Panels B and D show the results of the image analysis of two video recorded frames, with particles circled in red. Increased particle numbers in B, compared to C, match the higher density estimated found with the model.

Modelled particle concentration profiles were generated for two different days, matching the dates for which video image recordings were obtained (18^th^ and 19^th^ May, Fig 5). Panels A and C show the resultant density maps produced by the Lagrangian model for both dates, after a 48 hour run, starting from a uniform distribution of particles (see methods Section 6). Panels B and D show video-frames for each day with gelatinous zooplankton recorded at the same position (red dots in panels A and C). Panel B shows increased particle density, according to the Lagrangian map on the 18^th^; panel D shows lower densities for the 19^th^. Simulated vertical profiles and corresponding video frames (not shown here) also show higher densities in the upper part of the water column than below 40 m depth, as seen in Fig 4.

### Fish behaviour versus particle density map

For all of four sunfish GPS tracks, we investigated the relationship between fish behaviour and the simulated buoyant particle densities. Assuming that the simulated particles actually reflect the distribution of drifting zooplankton in the region, both sunfish FPT and speed (parameters used to determine the along the track ARS) were found to directly respond to estimated encountered prey-fields. Briefly, significant correlations (Spearman Rank Correlation) were obtained between log FPT and the respective locations’ averaged estimated density (*n* = 599 positions with FPT attributed) constrained to the tracking period (r_s_ = 0.184, *p*<0.001). Sunfish speed also decreased significantly with higher simulated densities encountered (r_s_ = -0.214, *p*<0.001, *n = 686*). Thus, with the estimation of high-resolution zooplankton densities we provide evidence for correlation between the behaviour of sunfish and areas of likely increased aggregation of simulated particles.

**Fig 6:**
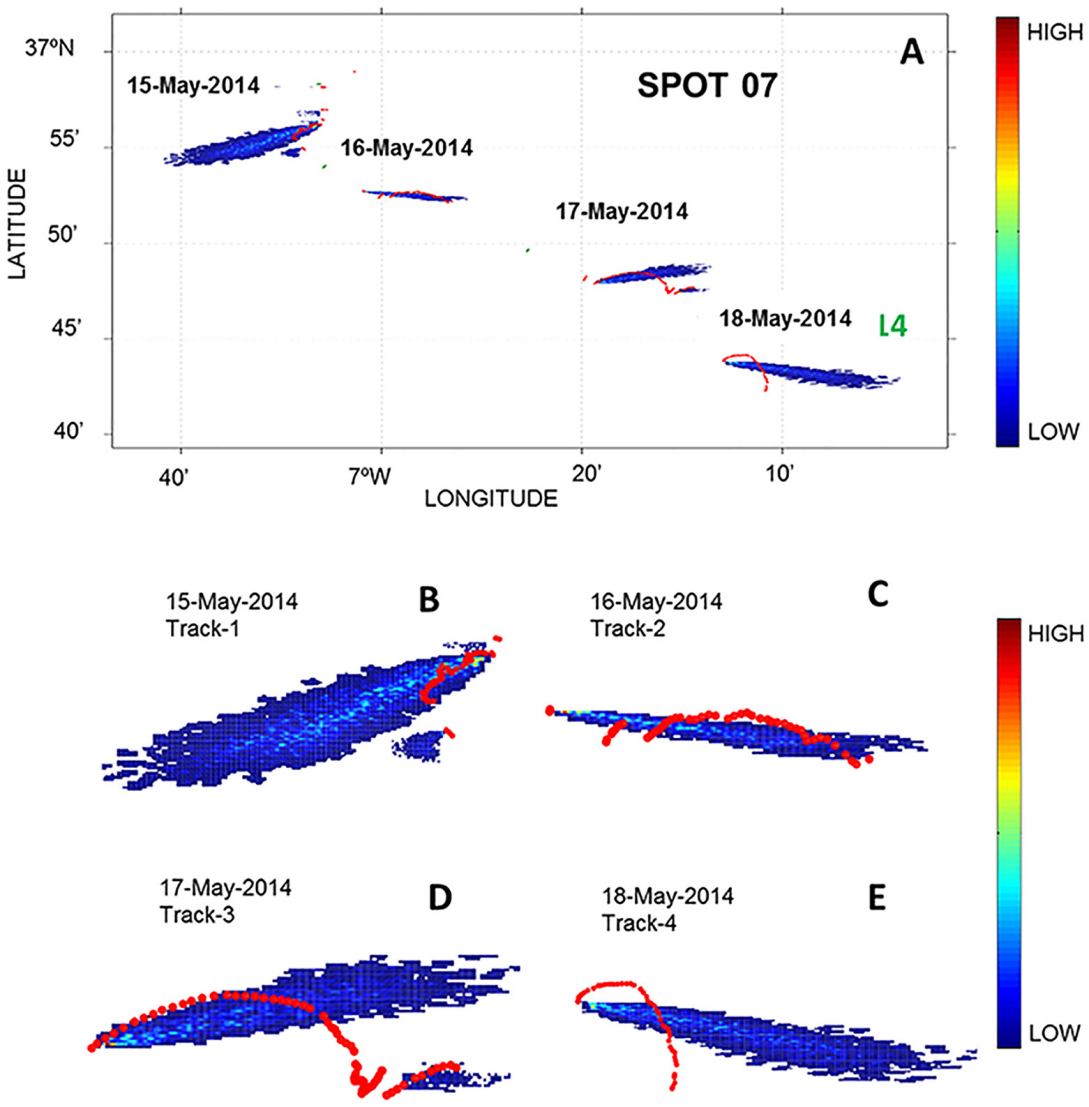
Location of tracks (red dots) and Lagrangian model output for SPOT-GPS-07. Panel A: particle density maps (colour bar) with fish tracks superimposed (red dots). Panel B-D: details of each track. The sunfish was tagged and released on May 15^th^ (track-1). The intervals without data correspond to their diving period. On May 18^th^ (track 4) the contact was lost after the last dive.

Lastly, by comparing the direction of fish movements with current headings obtained from CMEMS, we detected that sunfish do not always follow encountered currents. For example, sunfish tagged with SPOT-GPS 07 (Fig 6) was released on May 15^th^ and on its first day shows an erratic surface trajectory. At the end of this section (track-1) the sunfish dived and resurfaced the next day, May 16^th^ (track-2). The same pattern was repeated on May 17^th^ (track-3) and 18^th^ (track-4) (Fig 6, red dots). Lagrangian simulations were performed to check whether the fish tracks were aligned with the direction of the currents. At the beginning of each track (first position recorded for each day), Lagrangian particles were released in the model that was informed by current data obtained from CMEMS. By superimposing the particle density maps produced by the Lagrangian model (dark blue is low, red is high density–Fig 6), with the tracks, we were able to detect whether the fish was following the directionality of the currents. In track-1 the fish was swimming eastwards regardless of the current flow (Fig 6B); on track-3 (17^th^ May), however, the fish followed the mainstream current (Fig 6C). Since they appear to follow the current at times, we tested for differences in the magnitude of the currents during both days (15^th^ May with fish not following the current pattern *versus* 17^th^ May when the fish appears to swim with the flow). Importantly, stronger currents (mean = 0.15 ms^-1^ and variance of 0.0004 ms^-1^) were found for the 17^th^ compared to the 15^th^ (mean = 0.097 and variance of 0.0003 ms^-1^) and this difference in magnitude was statistically significant (*t* test = -16.08, df = 142 and *p* <0.001). These results suggest that the difference in current strength may explain the observed sunfish behaviour: in weak currents the fish will likely swim regardless of the direction of the features, but in strong currents they will likely be carried with the flow.

### Oceanographic settings

**Fig 7:**
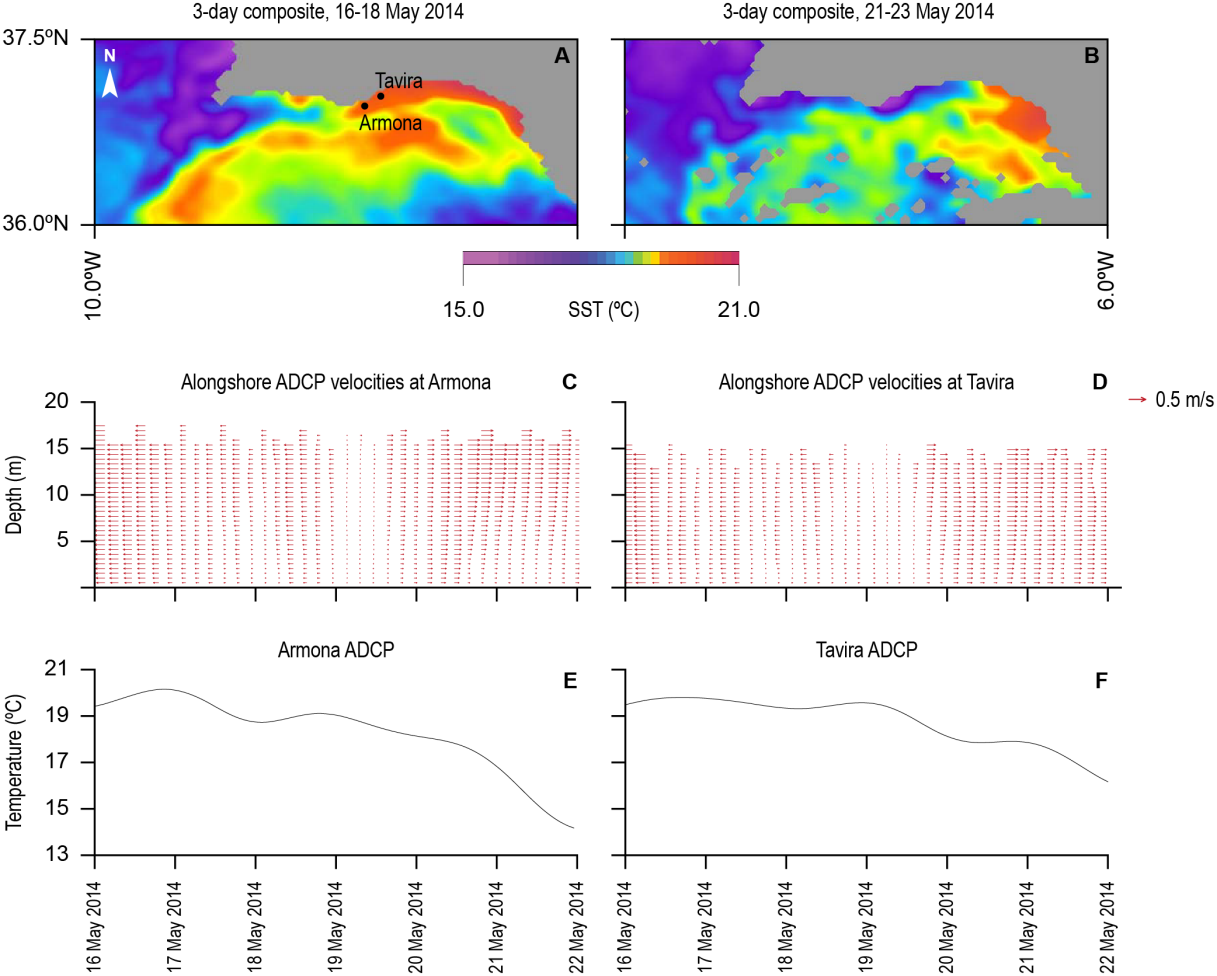
MODIS Aqua sea surface temperature composites for 16th-18th and 21st-23rd of May 2014 (a and b). The approximate locations of the Armona and Tavira ADCP’s are superimposed on the two composites. Alongshore (red vectors—vertical profiles plotted every 4 hours) and cross-shore (grey contours) ADCP velocities along the water column (c and d) at Armona and Tavira from the 16t^h^ to the 23^rd^ May 2014, capturing the inversion of the current. Temperature at the Armona and Tavira ADCP’s for the same period are displayed in e and f.

Our experiment started during the absence of upwelling, immediately followed by an upwelling event. The transition between the two contrasting patterns is depicted in Fig 7. The composite for 16^th^– 18^th^ May 2014 shows a warm water tongue invading the coastal region until about 8°W. The 21^st^– 23^rd^ May composite shows the opposed situation, with cold upwelled water occupying the coastal region and stretching into the eastern GoC. The establishment of the thermal fronts can be observed clearly. Although with opposed gradients, the location of the SST fronts are coarsely coincident in both patterns roughly over the shelf break, justifying in oceanographic terms, the higher occurrence of ARS in these region, as noted above in Section 2. The transition corresponds to the shift of the alongshore circulation from westward during the absence of upwelling to eastward during upwelling. The reversal of the alongshore currents was captured by the moored ADCP’s installed over the shelf, along with the drop in the water temperature at the bottom, of about 5°C in four days (Fig 7c–7f), consistent with the SST satellite images. The coastal circulation can be considered alongshore since it prevails over the cross-shore component by almost one order of magnitude, reaching speeds over 0.5 ms^-1^. The velocity data collected by the WG ADCP, confirms the prevalence of the eastward (positive) velocities in the region, peaking after 19^th^ May (Fig 7), in agreement with the coastal circulation associated with the upwelling event.

## Discussion

The major advance in this study was the use of operational robotic hardware that augments fine-scale tracking of ocean sunfish allowing for the characterisation of the near real-time environment explored by the fish. Although our study is limited in extension, it paves the way for future marine tracking developments. Briefly, the fine-scale tracking allowed to identify an increase in the time spent within a constricted region (ARS), in relation to other directed movements such as travel. The environmental integration of such behavioural patterns revealed that SST frontal regions play a major role in foraging, whereas Chl *a* revealed a negative correlation with fish foraging behaviour. Lastly, the hydrodynamic Lagrangian model, validated by both *in situ* WG data measurements and launched drifter trajectories, defined areas of likely accumulation of drifting particles. These were then used to inform Lagrangian zooplankton densities, which also correlated positively with these strong thermal gradients but not with Chl *a*. Altogether, SST fronts were detected as positive drivers for both fish foraging patterns and simulated prey abundance. Lastly, we show in the present study the combination of different disciplines and technologies to better understand important inter-species relationships, such as the predator-prey interactions.

### Tag performance

One problem associated with tracking systems is the location accuracy. For example, the lack of precision in Argos tags is likely to dominate, compared to the real movement made by the animal, whereas GPS tags have revealed details of migration routes and space use [14–16,47] or even the impact of currents on migration (e.g. [48]). The SPOT-GPS tag used in this study reflects higher position accuracy <10 m *versus* the 60 m associated with for instance the Fastloc-GPS^™^, which provides the best accuracy currently available for animal tracking technologies [7,49]. Not surprisingly, the geolocation estimates were several fold higher than those obtained by tracking sunfish with Argos tags (e.g. [34]). Our results demonstrate that these GPS transmitters have the robustness and efficiency needed in future marine species tracking studies, provided the species spends enough time at the surface. Compared to Fastloc-GPS^™^ for instance, which allows the rapid acquisition of GPS ephemeris when an animal surfaces, the GPS technology here employed demands that the animal spend longer periods at the surface (often at least 1 to 2 minutes depending on sea state). That said, the fact that a total of six GPS-tracked individuals did not report positions is likely to be attributed to fish behaviour (continuous diving with null surface times for satellite data retrieval), tag failure or fish mortality. Tag failures have been reported in different tracking systems, including Argos; the reasons behind transmitter signal loss can be identified as for instance, the depletion of batteries, water switch failure, antenna breakage, animal mortality or the premature detachment of tags [50]. However, no data was collected in this study, for these six fish, that allow us to discriminate between equipment failure and animal mortality.

### Environmental integration of sunfish behaviour

Sunfish are active normal diel vertical migrants (nDVM) with repeated daily dives below the thermocline [35,51–53] and these oscillatory movements have been linked to foraging behaviours [35,54]. In their study, Sims and collaborators detected along-the-path stopovers in GPS tracked sunfish movements, presumably to forage on encountered prey patches [13]. This behaviour was also depicted from the majority of GPS positions retrieved in our study, with track metrics such as FPT, speed and sinuosity informing ARS along the travelling motion of sunfish. Importantly, these movements are characteristic of a predator foraging in a profitable prey patch as residency of an animal is likely to increase with habitat profitability [55,56]. ARS can be detected along the performed path by consecutive smaller step-lengths in a more constrained region that results from increased turning rates at lower speeds as a response to increased intake [36]. Following [13], our analysis also revealed regions of increased search behaviour in between continuous motion along fish tracks, further confirming the utility of track metrics in the detection of behavioural patterns in tracking studies [36,57–60]. Notwithstanding, longer tracks (in the tens of days) would certainly provide better information on the sunfish behaviour and its relationship with the surrounding environment.

The integration of sunfish behaviour at fine-scales with the environment, confirmed a positive influence of both SST gradients and front intensity in this species’ search patterns. The broad movement of this species was already linked to sharp thermal variation both in the north-east Atlantic [34,61] and in the Pacific [51]. These frontal areas are known to be aggregation zones of buoyant organisms and lower trophic levels [62], thus attracting larger marine predators due to improved foraging opportunities [62]. The relationship among fronts and pelagic fish occurrence has been already established for swordfish [63], loggerhead turtles [64], sunfish [61], tuna and billfish [65] and shark species [4,66]. Hence, the clear association between sunfish ARS and thermal fronts further confirm the importance of such regions as productivity and biodiversity ‘hotspots’.

The contrary outcome was also found with regards to Chl *a*, with ARS occurring preferentially in areas of lower productivity. Not being a primary consumer, sunfish do not directly track primary productivity and thus the mismatch detected here between sunfish ARS and Chl *a* is probably a reflection of the actual mismatch between phytoplankton and zooplankton, previously described [67]. Despite the sparse literature on the diet of ocean sunfish [68], large individuals are known to feed predominantly on gelatinous zooplankton including salps, jellyfish and ctenophores [54,69,70]; smaller fish, recent studies reveal, also prey on benthic species [54,71]. Hence, sunfish do not actively pursue primary producers but respond to patchily distributed plankton [72].

With regards to the currents, tracked sunfish either moved along or against the directionality of these features. Previous studies have revealed that this species is an active swimmer independently of oceanic currents [13,52,73] and with daily average speeds recorded between 19.8 km/day and 26.8 km/day [13,51]. In the present study, sunfish movement in relation to currents were compared to those of a drifter launched at the same fish tagging location, which were in turn used to validate the Lagrangian model. We found that whereas the drifter followed a well-defined path eastward along the coast, similar to the upwelled water flow, sunfish tracks did not conform to the water motion.

### Zooplankton densities from drifting particles

In this study the *in situ* video recording of the water column encountered by the sunfish provided an estimation of drifted particles. These particles were recorded in a region without any direct river discharge, supporting the idea that the particles were mostly zooplankton specimens. Furthermore, and similarly to sunfish ARS environmental integration, both SST gradients and proximity of fronts played a key role in increased particle densities while SST and Chl *a* were again found to be negatively correlated with detected particle abundance. SST gradients or thermal fronts are known to be important biodiversity ‘hotspots’ due to increased nutrient mixing and retention which enhances the primary productivity [62]; whereas plankton tend to be entrapped in the convergent surface [74,75]. Subsequent aggregation of advected zooplankton drives the bottom-up processes associated with the trophic chain in the marine ecosystem [76,77]. In fact, the interaction between detritus, phytoplankton and zooplankton appear to determine the main trophic flow in the GoC [78]. In addition, prevailing GoC oceanographic structure, namely its persistent fronts, are known to enhance biodiversity in the region, e.g. the increased cephalopod paralarvae occurrence and dispersal [79]. Hence, using the video recorded particles as proxy for zooplankton is highly supportive. Moreover, the close link between sunfish foraging requirements and drifted particle density drivers, conform our hypothesis.

### Lagrangian models

During the present study we observed the transition between a non-upwelling phenomena to an upwelling event, off southern Iberian coast. In such conditions the geostrophic adjustment induces an eastward alongshore flow that advects cold upwelled water along the southern Iberian coast, until it encounters the warm water patch characteristic of the eastern Gulf of Cadiz. Such eastward coastal flow was captured in ADCP data (see Fig 7c and 7d, from 20th May onwards) and also confirmed by WG data. With these oceanographic settings a strong front between the upwelled and the warmer offshore waters was present in the region, suitable for the accumulation of particles. The observed current pattern was satisfactorily reproduced by the hydrodynamic model, completing the characterization of the region occupied by the tracked sunfish in terms of oceanographic currents while validating the output to feed the Lagrangian model. By using such models with drifters we simulated density maps of particles in the region at the surface and at three different depth layers. The vertical distribution of particles obtained from the AUV dives validated the model output by matching the simulated particles at different depths. Importantly, the higher concentrations of particles overlapped the main frontal area and steep thermal features where most sunfish ARS modes were traced.

Conventional wisdom on particle accumulation in frontal zones states that a set of physical and biological processes are responsible for enhanced productivity in fronts involving a physiological response of the organism to the physical dynamics. In convergent zones, buoyant particles remain at the surface despite weak downwelling vertical currents, leading to accumulation at fronts [74,80]. Frontal systems are highly variable in both space and time, with physical, chemical and biological factors changing across trophic levels [81]. By video recording the water column, the particle abundance was found to be markedly higher in well-mixed waters, which conforms to plankton distribution at depth. In stratified waters, it is expected that phytoplankton remain entrapped at the surface layers whereas zooplankton concentrate foraging in these regions of enhanced phytoplankton biomass [82]. On the other hand, well-mixed waters without a clear structure of either physical or biological factors do not create any boundary for the plankton’s vertical distribution. In coastal regions, with increased productivity it is expected to have an increased abundance of gelatinous zooplankton, as revealed by the results of Lilley et al., [83]. This further confirms the mismatch previously described for both phytoplankton and zooplankton [67] and likely reflects the lack of association found between both zooplankton densities and sunfish ARS with Chl *a*.

These findings further support previous studies where the importance of fronts as preferential pelagic conservation areas was raised [84], with emphasis towards reducing pressure on fisheries. Sunfish habitat preferences in relation to frontal regions has been described for several distinct regions [34,51,61] and these frontal regions are regularly under spatially explicit anthropogenic threats, such as intense fishery activity [4,62]. In fact, worldwide elevated sunfish bycatch records occur in specific frontal regions, such as the California Current [51,52].

### Oceanography—persistent fronts as a particle accumulation areas

The northern margin of the GoC, is a region characterised by intense primary productivity associated with a strong mesoscale activity induced by a wide range of forcing mechanisms [85–88]. The region is dominated by upwelling events during the summer season, roughly defined from March/April to September/October [89], driven by northerly/westerly winds on the west/south Iberian coast and the development of an associated geostrophic current flowing eastward along the northern margin of the GoC. This flow is continuous from the western Iberian coast and possibly feeds the inflow of Atlantic water into the Mediterranean (see S2 Fig). These upwelling regimes are interleaved by periods when warm waters originating in the eastern part of the GoC invade the coastal region [85,89,90]. This second regime corresponds to the absence of upwelling in the region due to wind relaxation. It corresponds to the westward progression of a warm and saline coastal counter-current progressing from the eastern GoC. The thermal contrast between the different waters occupying the continental shelf and the offshore waters promote the occurrence of strong frontal regions and enhance the primary productivity. The interaction of the coastal counter-current with the upwelling jet results in conspicuous mesoscale features over the continental shelf and slope with the consequent formation of strong thermal fronts. The irregular bottom topography, along with the coastline configuration, modulates the spatial development of the fronts. Thus, the occurrence of thermal fronts, roughly located over the shelf break, is a persistent characteristic of the region, whatever the prevailing oceanographic regime. Hence, although subject to seasonal and interannual variation [85], this frontal area in the GoC may be a major driver for the persistent local occurrence of sunfish [34]. In the latter study, the authors suggest that the high production in the coastal region of the GoC supports a nursery area for younger stages of sunfish, by providing enhanced feeding opportunities that can support fast growth rates.

In summary, by GPS tracking four individuals at the fine-scale, our study provides for the first time, to our knowledge, a fine scale description of the environmental preferences underpinning both sunfish foraging and likely its preferred prey concentration drivers. We identified foraging behaviours of sunfish and related them with the local environment, disclosing important links with steep thermal gradients. Furthermore, *in situ* buoyant particles (as proxy for zooplankton) were also correlated with similar frontal regions preferences. Altogether, this work presents the successful combination of several different technologies (satellite transmitters, underwater and surface autonomous vehicles, hydrodynamic modelling) in the environmental integration of marine animal movement and behaviours. Lastly, disseminating the technology out to a broader non-scientific audience was part of the experimental goal. The technological setting allowed not only real-time commanding of the vehicles *in situ*, but shore-side situational awareness for educational purposes with middle-school students tracking tags and vehicles in near real-time in their classroom, while interacting with scientists in the field via video-conference.

## Supporting Information

S1 Fig Northward (left) and Eastward (right) daily component provided by the Wave Glider (line) and model output (dots) averaged over two days and registered at 18 m depth. (TIF)

S2 Fig Example of a drifter track superimposed on a MODIS SST map composite from May 25^th^ to June 1^th^ (top) and June 2^th^ to 9^th^ June (bottom), both 2014. (TIF)

S3 Fig a) Example of a drifter (red line, with dates) and particles (blue lines) tracks, b) Atlantic section of the drifter track before entering the Mediterranean Sea (red line) and mean trajectory of the 200 particles released in the Lagrangian model (green line). (TIF)

S1 File Archive of Drifter data. (ZIP)

S2 File Archive of AUV tracks data. (ZIP)

S1 Text Supplementary Material for integrated monitoring of *Mola mola* behaviour in space and time. (DOCX)
