# Supplementary figures and images for: Integrated Monitoring of Mola mola Behaviour in Space and Time
Source: PLoS One. 2016 Aug 5;11(8):e0160404. doi: 10.1371/journal.pone.0160404 (PMC4975458; doi:10.1371/journal.pone.0160404)

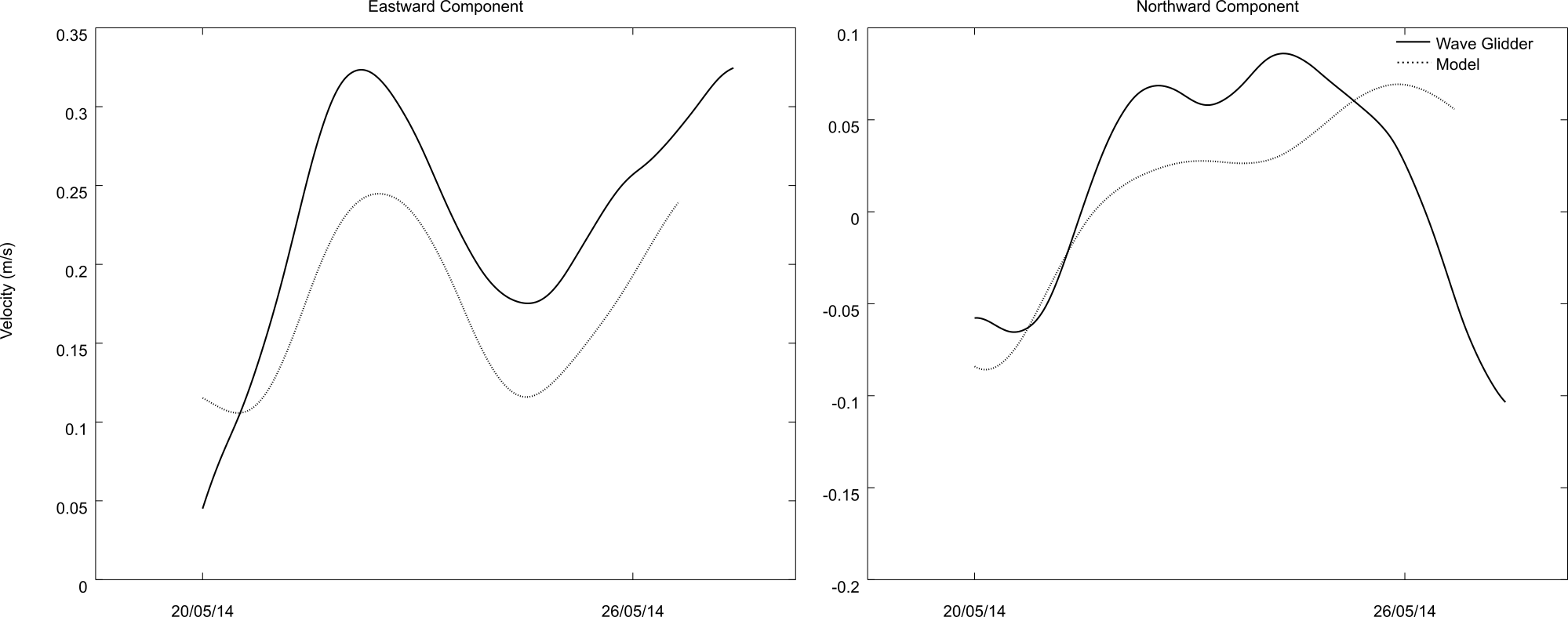

Supplement: S1 Fig — (TIF) [file pone.0160404.s001.tif]

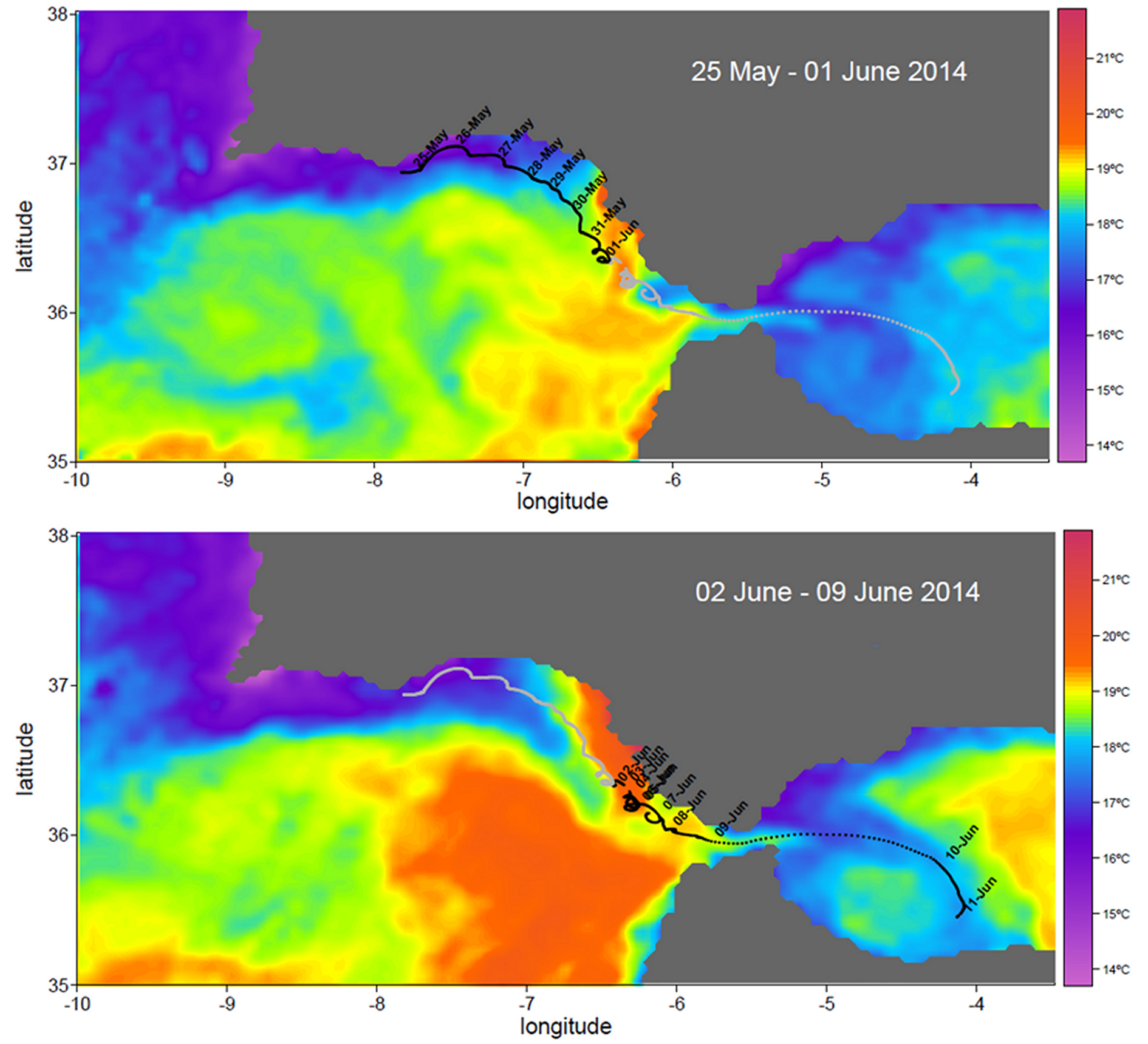

Supplement: S2 Fig — (TIF) [file pone.0160404.s002.tif]

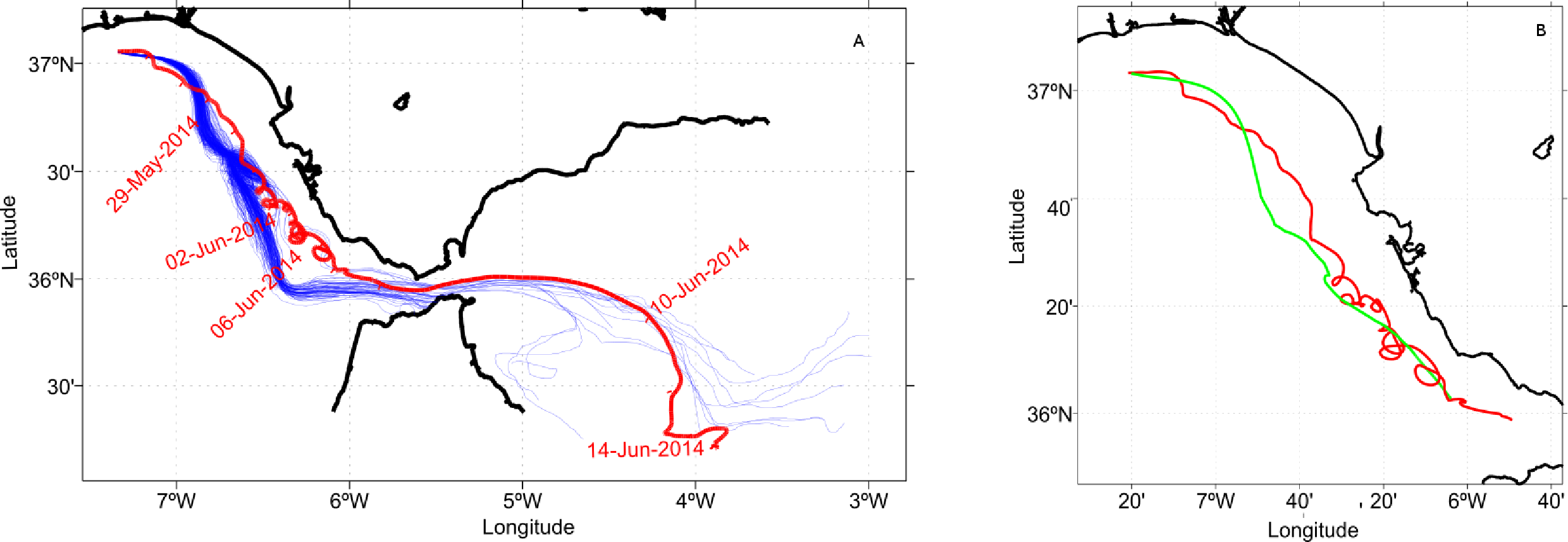

Supplement: S3 Fig — (TIF) [file pone.0160404.s003.tif]
