# Supplementary material for: Integrated Monitoring of Mola mola Behaviour in Space and Time
Source: PLoS One. 2016 Aug 5;11(8):e0160404. doi: 10.1371/journal.pone.0160404 (PMC4975458; doi:10.1371/journal.pone.0160404)
Supplement: S1 Text — (DOCX) [file pone.0160404.s006.docx]

**Supplementary Material for:**

**Integrated monitoring of *Mola mola* behaviour in space and time**

Lara L. Sousa^1,7^, Francisco López-Castejón^2^, Javier Gilabert^2^, Paulo Relvas^3^, Ana Couto^1^, Nuno Queiroz^1^, Renato Caldas^4^, Paulo Sousa Dias^4^, Hugo Dias^4^, Margarida Faria^4^, Filipe Ferreira^4^, António Sérgio Ferreira^4^, João Fortuna^4^, Ricardo Joel Gomes^4^, Bruno Loureiro^4^, Ricardo Martins^4^, Luis Madureira^5^, Jorge Neiva^4^, Marina Oliveira^4^, João Pereira^4^, José Pinto^4^, Frederic Py^4^, Hugo Queirós^4^, Daniel Silva^4^, P.B. Sujit^4,8^, Artur Zolich^6^, Tor Arne Johansen^6^, João Borges de Sousa^4^, Kanna Rajan^4,6*^

**Details of the adaptation of tags for this experiment and validation of the hydrodynamic and Lagrangian models are described in this supplemental material**

1. Adaptation of commercial personal trackers locators to fish tags.

The SPOT-GPS tags were based on the SPOT® personal tracker. A single-board microcontroller was developed so the tracker could be switched on and off by bypassing the control buttons used by human operators. The on-off signal was generated by a simple electric circuit that remained closed when the tag was underwater. All the electronics were contained in a 5cm diameter X 8.7cm long cylinder of Polyurethane Foam to increase buoyancy. A mixture of Polyurethane Resin and hollow glass microspheres were used to decrease the density of the mixture without compromising the resistance of the material for casting the tag. To improve the hydrodynamic performance the front of the tag had spherical shape. Before deployment, tags were tested for pressure tolerance in a hyperbaric chamber at 60 bar ensuring that tags could operate in up to 600 meters of depth. The electronics are powered by a small battery pack, which also serves as a ballast to keep the tag upright while at the surface, enabling the antennas to be kept out of the water for radio transmission. Since the ballasting provided by the batteries alone is inadequate, we used a combination of flotation and ballasting components.

2. The WaveGlider

The WaveGlider (WG) is a wave-energy based autonomous surface vehicle from Liquid Robotics, Sunnyvale, California. It consists of a two-body system with a surface expression with all electronics and solar panels to power an on-board battery, and a subsurface glider with slats which powers the vehicle’s forward movement. The vertical potential wave energy is converted into horizontal movement by these slats attached to the glider, which in turn is connected to the surface with an umbilical tether. The glider is typically 7 meters below the surface. The vehicle uses a graphical user interface available to those on the field, which can be used to communicate a set of desired patterns and detailed waypoint information. 24x7 operators in California, using the on-board Iridium modem, command the vehicle while data in near real-time is collected and stored for user access. For the experiment, the WG was operated independently of the AUV command and control infrastructure.

3. Model validation

3.1. Current field validation with the WG data

*In situ* data recorded by the ADCP mounted on the WG were used to validate the currents maps provided by Copernicus Marine Environment Monitoring Service (CMEMS).

Validation of local hydrodynamics was checked to verify whether the sunfish follow the currents or swim against them. S1 Fig shows a comparison between the Northward and Eastward current component (*u* and *v*) respectively at 18 m depth provided by the WG and the hydrodynamic model output. The Pearson correlation coefficient (*r*) and pbias for Northward (*r* = 0.99, pbias=22) and Eastward component (*r*= 0.75, pbias = 20) showed a good performance fit of the model.

S1 Fig. Northward (left) and Eastward (right) daily component provided by the Wave Glider (line) and model output (dots) averaged over two days and registered at 18 m depth.

3.2. Lagrangian model validation with drifters

To validate the Lagrangian model three SPOT-based drifters were specifically built to report positions every 15 minutes. To ensure the drifters were dragged by underwater currents, a drogue at 3 meters depth was installed and suspended. Drifters were deployed at the same location where the sunfish were tagged and released. Drifter tracks can be found in S1 Zip file.

S2 Fig. Example of a drifter track superimposed on a MODIS SST map composite from May 25^th^ to June 1^th^ (top) and June 2^th^ to 9^th^ June (bottom), both 2014.

The SST map shows that an upwelling situation induced by favourable winds was established along the western coast of Portugal and the northern margin of the GoC. A geostrophic current associated with the upwelling mechanism advects cold upwelled water eastward along the coast, until it encounters the warm water patch at -6.5 W by the end of May. This current constitutes the main inflow of North Atlantic Central Water (NACW) into the Mediterranean. As it crosses the Strait of Gibraltar, it accelerates and curls anticyclonically in the Alboran Sea. In the following week the warm patch spreads westward along the coast and the cold upwelled water and associated current are pushed offshore.

Validations of the Lagrangian model based on drifter trajectories have inherent errors, due to instabilities in the current field that can change the drifter path reducing the Lagrangian predictability (91). To reduce this error and obtain a representative trajectory of the current field we have used the current fields provided by CMEMS, which are a daily averaged field of hourly hydrodynamic outputs from the Nemo model. We used this daily averaged map to force our Lagrangian model where we released 200 virtual particles at the time and location where drifters were deployed. The Lagrangian model had a time step of 1 hour, implying that one particle moved from position A (x_a_,y_a_,z_a_) at time 0 (t_0_) to position B (x_b_,y_b_,z_b_) 1 hour later (t_1_). The particle at position B (x_b_,y_b_,z_b_) was in turn, forced to displace by the current at this point extracted from the daily averaged field of currents to C (x_b_,y_b_,z_b_) next hour (t_2_). This step was repeated after 24 hours when a new averaged field of currents was delivered by CMEMS. Following the methodology described in [92] we spatially averaged the position of the 200 particles at any time step (green line in S3 Fig a.) to obtain a maximum distance between the drifter and the mean particle trajectory of 19 km, well within the range described in [92,93]. Only the Atlantic branch of the track was used as many Lagrangian particles did not cross the Gibraltar Strait, thereby increasing the error in the Mediterranean Sea area.

S3 Fig a) Example of a drifter (red line, with dates) and particles (blue lines) tracks, b) Atlantic section of the drifter track before entering the Mediterranean Sea (red line) and mean trajectory of the 200 particles released in the Lagrangian model (green line)

The drifter track that was tracked for a longer period of time, is consistent with this oceanographic pattern described above. It follows a well-defined path from the 25^th^ to the 31^st^ of May to be trapped until June 4^th^ in the eddy field formed in the frontal region between the warm coastal counter current and the opposed cold water current upwelled further to the west. Finally, on the June 8^th^, the drifter was released from the vortices field and accelerated to enter the Mediterranean Sea turning southward into the Alboran Sea. Estimated mean velocities of the drifter are: 0.25 m/s from the 25^st^ to the 31^st^ May, 0.17 m/s from June 1^st^ to 8^th^ - progressing only 80 to 90 km towards the Strait due to the contorted path and almost 0.78 m/s from the 8^th^ to the 10^th^ of June, with a peak mean velocity of 1.16 m/s during June 9^th^. The Lagrangian model, fed with the output of the hydrodynamic model, shows a small possibility of a particle entering the Mediterranean Sea, since they are easily trapped in eddies off the south of Cadiz.

**References**

92. Barron CN, Smedstad LF, Dastugue JM, Smedstad OM. Evaluation of ocean models using observed and simulated drifter trajectories: Impact of sea surface height on synthetic profiles for data assimilation. Journal of Geophysical Research. 2007;112(C7): C07019.

93. Mantovanelli A, Heron ML, Prytz A, Steinberg CR, Wisdom D, editors. Validation of radar-based Lagrangian trajectories against surface-drogued drifters in the Coral sea, Australia. MTS/IEEE OCEANS 2011;19-22 Sept. 2011
